# A scoping review and mapping exercise comparing the content of patient-reported outcome measures (PROMs) across heart disease-specific scales

**DOI:** 10.1186/s41687-019-0165-7

**Published:** 2020-01-23

**Authors:** Beatrix Algurén, Michaela Coenen, Dan Malm, Bengt Fridlund, Jan Mårtensson, Kristofer Årestedt, on behalf of the Collaboration and Exchange in Swedish cardiovascular caring Academic Research (CESAR) group

**Affiliations:** 1 Department of Food and Nutrition, and Sport Science, Faculty of Education, University of Gothenburg, PO Box 300, 405 30 Gothenburg, Sweden; 2 The Jönköping Academy for Improvement of Health and Welfare, School of Health Sciences, Jönköping University, Jönköping, Sweden; 3 Institute for Medical Information Processing, Biometry and Epidemiology (IBE), Chair of Public Health and Health Services Research, Research Unit for Biopsychosocial Health, LMU Munich, Munich, Germany; 4 Pettenkofer School of Public Health, Munich, Germany; 5 ICF Research Branch, a cooperation partner within the WHO Collaborating Centre for the Family of International Classifications in Germany (at DIMDI)*, Nottwil, Switzerland; 6 Department of Nursing, School of Health and Welfare, Jönköping University, Jönköping, Sweden; 7 Centre of Interprofessional Collaboration within Emergency care (CICE), Linnaeus University, Växjö, Sweden; 8 Faculty of Health and Life Sciences, Linnaeus University, Kalmar/Växjö, Sweden; 9 The Research Section, Region Kalmar County, Kalmar, Sweden

**Keywords:** International Classification of Functioning, Disability and Health, Heart disease, Healthcare quality, Patient outcome assessment, Patient-reported outcome measures, Person-centered, Review

## Abstract

**Background:**

Over the past decade, the importance of person-centered care has led to increased interest in patient-reported outcome measures (PROMs). In cardiovascular care, selecting an appropriate PROM for clinical use or research is challenging because multimorbidity is often common in patients. The aim was therefore to provide an overview of heart-disease specific PROMs and to compare the content of those outcomes using a bio-psycho-social framework of health.

**Methods:**

A scoping review of heart disease-specific PROMs, including arrhythmia/atrial fibrillation, congenital heart disease, heart failure, ischemic heart disease, and valve diseases was conducted in PubMed (January 2018). All items contained in the disease-specific PROMs were mapped to WHO’s International Classification of Functioning, Disability and Health (ICF) according to standardized linking rules.

**Results:**

A total of 34 PROMs (heart diseases in general *n* = 5; cardiac arrhythmia *n* = 6; heart failure *n* = 14; ischemic heart disease *n* = 9) and 147 ICF categories were identified. ICF categories covered *Body functions* (*n* = 61), *Activities & Participation* (*n* = 69), and *Environmental factors* (*n* = 17). Most items were about experienced problems of *Body functions* and less often about patients’ daily activities, and most PROMs were specifically developed for heart failure and no PROM were identified for valve disease or congenital heart disease.

**Conclusions:**

Our results motivate and provide information to develop comprehensive PROMs that consider activity and participation by patients with various types of heart disease.

## Background

Heart disease is a common and increasing health problem worldwide [1]. Independent of diagnosis and etiology, heart diseases usually have a significant negative impact on people’s quality of life (QoL) and well-being with high symptom burden, emotional reactions, reduced physical capacity, and social isolation [2–6]. Over the past decade, the importance of person-centered care, which includes patients’ experiences of diseases and their impact on their daily lives, has been recognized [7–10]. Despite the fact that different definitions of “centeredness” exist within the literature (e.g. “patient”, “client”, or “customer”), they have three overarching common themes: a) an understanding of the person and their lived experience of health conditions and disability by taking a holistic view of health with recognition of psycho-social factors beyond just bodily functions and anatomical structures, b) patient empowerment in decision making, and c) the creation of relationships in care and treatment and the promotion of trust formed by continuity and coordination [11]. Understanding patients’ experiences of health conditions and disability and those issues that are important but less obvious to healthcare professionals can be assessed through patient-reported outcome measures (PROMs). PROMs have been defined as “… any report of the status of a patient’s health condition that comes directly from the patient (i.e., without the interpretation of the patient’s responses by a physician or anyone else)” [12] (p1). Thus, PROMs can be seen as an umbrella term for self-rating instruments that measure constructs such as health, health-related states, health-related QoL, well-being, and symptoms among other things [13], and they have been shown to be an important aspect of healthcare quality and value-based healthcare [14–17].

Selecting an appropriate PROM for clinical use or research is challenging not least because multimorbidity is common in patients with cardiovascular disease [18]. For example, a common sequela of multimorbidity is the co-existence of heart failure, atrial fibrillation, and hypertension, and these are often seen in combination with depression and chronic kidney disease [19]. The advantage of disease-specific versus generic PROMs are that disease-specific PROMs are more specific and sensitive to capture the distinctive problems that patients with a specific health condition experience. In case of multimorbidity, one disease-specific PROM might not portray the broad range of experienced problems and disease-specific PROMs for each morbidity should be used instead. However, this will increase the risk for content overlapping and thereby increase respondent’s burden without adding new information. In order to facilitate the selection of proper disease-specific PROMs or even only a generic set of important items for patients with various heart diseases, there is a need to analyze and compare existing instruments in order to get a better understanding of their content (i.e. items). For describing and comparing health information from outcome measures across diagnoses, the International Classification of Functioning, Disability and Health (ICF) from the World Health Organization (WHO) has been identified as a useful tool [20]. The ICF provides a common framework and a neutral language to describe health and health-related states from a bio-psycho-social perspective and comprises *Body functions*, *Body structures*, *Activities & Participation*, *Environmental factors*, and *Personal factors* [21]. The overall aim of this literature review was to provide an overview of heart-disease specific PROMs and to compare the content of those outcome measures using the ICF as a framework. This knowledge is of importance for clinicians and researchers in order to select appropriate PROMs for different kinds of care, treatments, clinical settings, and research purposes. It will give a better understanding of the common aspects of PROMs in the field of heart diseases from a bio-psycho-social perspective as well as identify what aspects are missing and need further development.

## Methods

### Design

A scoping review was conducted to get an overview of all existing disease-specific PROMs for patients with heart diseases, including heart diseases in general, ischemic heart disease, heart failure, arrhythmia, valve disease, and/or grown-up congenital heart disease [22]. The items of the identified PROMs were compared using the ICF as a framework.

### Scoping review of disease-specific PROMs

**Fig. 1:**
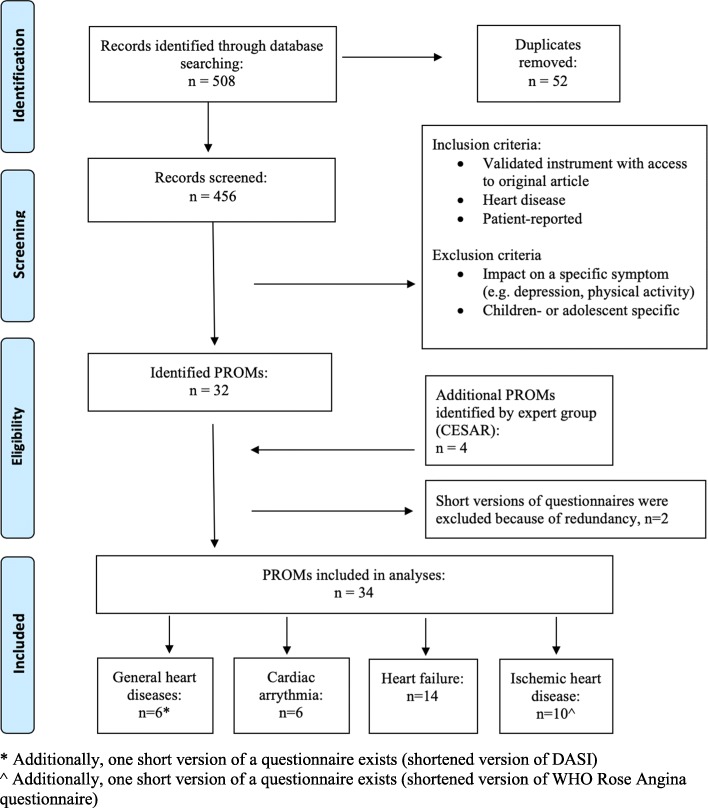
Flow diagram of the literature search in PubMed, conducted in January 2018

The systematic literature search was carried out in PubMed. A series of pilot searches were conducted during 2017 to identify relevant search terms. The pilot searches were discussed with an university librarian with special competence in medical science and literature searches. Based on this the following search terms and truncations were identified; questionnaire*, valid*, scale*, index*, develop*, heart disease*, ischemic heart disease*, angina*, myoc*, heart failure, arrhythmia, atrial, valve heart disease*, and congenital heart disease*. Together with the librarian, we also constructed multiple search combinations of these terms for each cardiac diagnose, with restrictions to the title (Additional file 1). The final search was conducted in January 2018 and was limited to English articles, while no limitations were used for publication type or year. Abstracts identified in the search were screened and selected based on the following two inclusion criteria: (1) PROMs developed or modified specifically for patients with heart disease in general or ischemic heart disease, heart failure, arrhythmia, valve diseases, and congenital heart disease and (2) some kind of validation process. Exclusion criteria were the following: PROMs developed for children and adolescent (aged under 18 years), PROMs measuring single symptoms or domains (e.g. fatigue, knowledge, risk behaviors, or locus of control), and patient-reported experience measures (e.g. measurements of treatment satisfaction). In total, 32 PROMs were identified, including short versions of two questionnaires. Additionally, an expert consultation was conducted. In doing so, four PROMs were identified by the expert group – the Collaboration and Exchange in Swedish cardiovascular caring Academic Research (CESAR) network (www.cesar-network.com). In total, 34 PROMs were included in the present mapping exercise, excluding the short versions of two instruments (Fig. 1).

### Content comparison of disease-specific PROMs

All items contained in the identified 34 disease-specific PROMs were mapped to the ICF according to standardized and established linking rules [23–25] first by identifying linking units in the items and second by mapping these linking units to the most specific ICF category (1st to 4th level of classification). A linking unit is a piece of information with the same meaning and often correspond to one item of a questionnaire. For example, one question in the Patient Perspective of Arrythmia Questionnaire (PPAQ) is ‘In the past 4 weeks, how often on average did your fast heart rhythm occur?’, where ‘*heart rhythm’* is the specific linking unit and b4101 (heart rhythm) the corresponding ICF category. Another example from the same questionnaire is the question ‘During the past 4 weeks, how many days did you miss work or school due to your fast heart rhythm?’, with one linking unit ‘*missing work’* linked to d850 (Remunerative employment) and the second is ‘*missing school*’ linked to d820 (School education). If a linking unit was not addressed by the ICF, the option “not covered” was chosen (e.g. “Having a chance for a happy future” or “Family’s health”). Concepts referring to *Personal factors* were mapped to this component of the ICF (pf – “Satisfaction with oneself in general”). However, because *Personal factors* are not further classified, no specific ICF codes were assigned and thus were not included in the following tables and figures.

Identification of linking units and mapping (“linking”) to the ICF was performed independently by two researchers experienced in the application of the ICF linking rules (BA, physical therapist; MC, psychologist). Consensus between the two researchers was achieved to decide which ICF category should be linked to each linking unit. In case of disagreement, the arguments were discussed until consensus on the final category was reached. Descriptive statistics were performed to provide absolute and relative frequencies of linked ICF categories.

## Results

### Disease-specific PROMs for heart diseases

Table 1 gives an overview of the identified instruments. In total, 34 PROMs were found by the scoping review and expert consultation, but the Duke Activity Status Index (DASI) and WHO Rose Angina questionnaire also exist as short versions and these versions were not explicitly analyzed in the present study. Of all identified PROMs, 5 are designed for use in patients with heart diseases in general, 6 for patients with cardiac arrhythmia, 14 for patients with heart failure, and 9 for patients with ischemic heart disease. No PROM was found for valve diseases or for congenital heart disease.

**Table 1:** Overview of the heart-disease specific PROMs identified by the systematic literature search (*N* = 34)

Name of PROMs	Abbreviation	Reference
Heart diseases in general (*n* = 5)		
Duke Activity Status Index (Duke Activity Status Index – short)	DASI (DASI – short)	Hlatky MA et al., 1989 [26] (Alonso J et al., 1997 [27])
HeartQoL Questionnaire	HeartQoL	Oldridge N et al., 2013 [28]
Multidimensional Index of Life Quality^a^	MILQ	Avis NE et al., 1996 [29]
Quality of Life Index - Cardiac version - IV	QLI cardiac version	Ferrans C & Powers M, 1985 [30]
Quality of Life Instruments for Chronic Diseases - coronary heart disease	QLICD-CHD	Wan et al., 2014 [31]
Cardiac arrythmia (*n* = 6)		
AF-QoL Questionnaire	AF-QoL-40	Badia X et al., 2007 [32]
Arrhythmia-specific Questionnaire in Tachycardia and Arrhythmia	ASTA	Walfridsson U et al., 2012 [33] Walfridsson U et al., 2015 [34]
Atrial Fibrillation	AF6	Härdén M et al., 2009 [35]
Atrial Fibrillation Effect on Quality-of-Life Questionnaire	AFEQT	Spertus J et al., 2011 [36]
Patient Perception of Arrhythmia Questionnaire	PPAQ	Wood KA et al., 2009 [37]
Quality of Life Symptom based for Atrial Fibrillation Questionnaire	QLAF	Braganca EO et al., 2010 [38]
Heart failure (*n* = 14)		
Cardiac Health Profile of Congestive Heart Failure	CHPchf	Mannheimer B et al., 2007 [39]
Care-Related Quality of Life survey for Chronic Heart Failure	CaReQoL CHF	Van Kessel P et al., 2017 [40]
Chronic Heart Failure Questionnaire	CHQ	Guyatt GH et al., 1989 [41]
Congenital Heart Disease-TNO/AZL Adult Quality of Life^a^	CHD-TAAQOL	Kamphuis M et al., 2004 [42]
Fragebogen zur Erfassung körperlichen Wohlbefindens	FEW16	Kolip P et al., 1999 [43]
Heart Valve Disease Impact on daily life	IDCV	Padilha et al., 2007 [44]
Heart Failure Somatic Awareness Scale	HFSAS	Jurgens CY et al., 2006 [45]
Kansas City Cardiomyopathy Questionnaire	KCCQ	Green CP et al., 2000 [46]
Left Ventricular Dysfunction Questionnaire	LVD-36	O’Leary CJ &Jones PW, 2000 [47]
Memorial Symptom Assessment Scale-Heart Failure^a^	MSAS-HF	Zambroski CH et al., 2005 [48]
Minnesota Living with Heart Failure Questionnaire	MLHF	Rector TS et al., 1987 [49]
Quality of Life Questionnaire in Severe Heart Failure	QLQ-SHF	Wiklund I et al., 1987 [50]
Symptom Status Questionnaire - Heart Failure	SSQ-HF	Heo S et al., 2014 [51]
Traditional Chinese Medicine inquiry	TCM inquiry	Fu TC et al., 2016 [52]
Ischemic heart disease (*n* = 9)		
Angina-related Limitations at Work Questionnaire	ALWQ	Lerner DJ et al., 1998 [53]
Angina Pectoris Quality of Life Questionnaire	APQLQ	Marquis P et al., 1995 [54]
Cardiovascular Limitations and Symptoms Profile	CLASP	Lewin RJ et al., 2002 [55]
Myocardial Infarction Dimensional Assessment Scale	MIDAS	Thompson DR et al., 2002 [56]
Quality of Life Index	QLI	Rukholm E, et al., 1994 [57]
Quality of Life Questionnaire	QLMI-2	Valenti L, et al., 1996 [58]
Seattle Angina Questionnaire	SAQ	Spertus JA et al., 1995 [36]
Summary Index for the Assessment of Quality of Life in Angina Pectoris^a^		Wilson A et al., 1991 [59]
WHO Rose Angina Questionnaire (Shortened WHO Rose Angina Questionnaire)		Rose GA, 1962 [60] (Lawlor DA et al., 2003 [61])

*PROMs* Patient-reported outcome measures

^a^Identified by the expert group

### Content of PROMs from a bio-psycho-social perspective

In total, the items of the PROMs could be mapped to 147 ICF categories covering 61 *Body functions*, 69 *Activities & Participation*, and 17 *Environmental factors* (see Additional files 2, 3 and 4). The PROMs that included most aspects of functioning were the Patient Perception of Arrhythmia Questionnaire (PPAQ) (27 ICF categories), the Memorial Symptom Assessment Scale-Heart Failure (MSAS-HF) (26 ICF categories), and the Summary Index for the Assessment of Quality of Life in Angina Pectoris (25 ICF categories) (Table 2). These included items that covered *Body functions* such as “Temperament and personality functions” (b126) but as well *Activities & Participation* such as “Recreation and leisure” (d920). Overall, *Body functions* were most frequently asked about and were included in all 34 PROMs, while 28 PROMS addressed *Activities & Participation*, 16 addressed *Environmental factors*, and 12 addressed *Personal factors.*

**Fig. 2:**
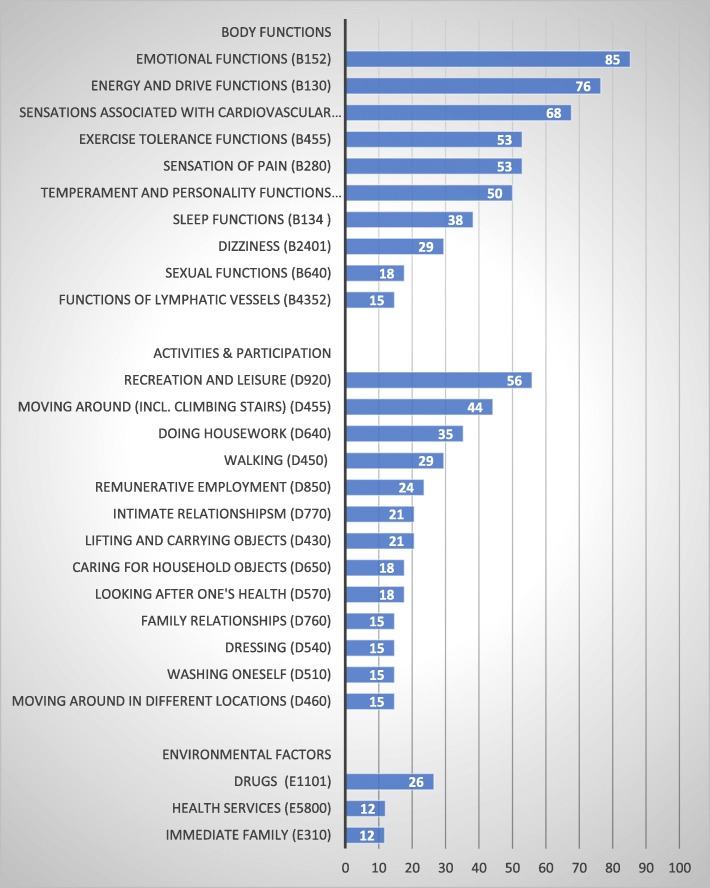
Most frequently mapped ICF categories (in percent) across the 34 PROMs with the results stratified by *Body functions*, *Activities & Participation*, and *Environmental factors*

Table 2 and Fig. 2 give an overview of the content of the PROMs.

**Table 2:**
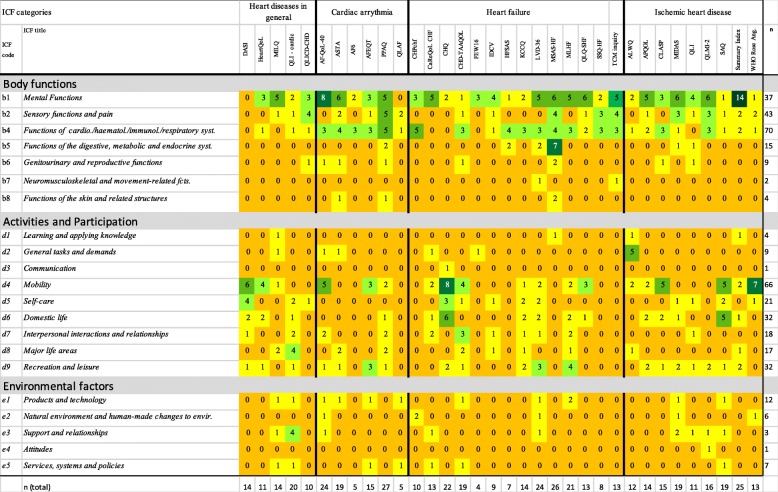
Overview of the number of different aspects measured by each PROM (*n*=34) linked to ICF chapters

The numbers represent how many questions on each questionnaire addressed the respective ICF chapter. Darker colors present higher number of questions

#### Body functions

All PROMs besides the DASI, which addressed only *Activities & Participation*, covered at least one body function. In total, 61 ICF categories were identified. The most frequently linked category was “Emotional functions” (b152), which was addressed in 29 (85%) PROMs, and this was followed by “Energy level” (b1300) (*n* = 26, 77%), and “Sensations associated with cardiovascular and respiratory functions” (b460) (*n* = 23, 68%) (Additional file 2 and Fig. 2). “Exercise tolerance functions” (b455), “Sensation of pain” (b280), and “Temperament and personality functions” (b126) were included in half of the PROMs.

#### Activities & participation

Thirty out of the 34 PROMs covered aspects referring to *Activities & Participation*. The six PROMs that did not covered any aspects of *Activities & Participation* at all were Atrial Fibrillation (AF6), the Quality of Life Symptom based for Atrial Fibrillation Questionnaire (QLAF), the Cardiac Health Profile of Congestive Heart Failure (CHPchf), the Heart Failure Somatic Awareness Scale (HFSAS), the Symptom Status Questionnaire - Heart Failure (SSQ-HF), and the Traditional Chinese Medicine inquiry (TCM inquiry). In total, 69 categories were identified, and the most frequently mapped categories were “Recreation and leisure” (d920), which includes sports and running and was addressed in 19 instruments (56%), “Moving around” (d455), which includes climbing stairs and was addressed in 15 PROMs (44%), and “Doing housework” (d640), which was addressed in 12 PROMs (35%) (Additional file 3 and Fig. 2).

#### Environmental factors and personal factors

Almost half of the PROMs (*n* = 16) addressed *Environmental factors*. In total, 15 categories were identified, with the most frequently linked categories being “Drugs” (e1101), which was addressed in 9 (27%) PROMs and “Immediate family” and “Health services” (e310 and e5500), which were both addressed by 4 (12%) PROMs (Additional file 4 and Fig. 2). Twelve of the 34 PROMs included one or two questions addressing *Personal factors* (the most common were, for example, Being bothered by having problems, Having difficulties relaxing, and Feeling like a burden to the family, with the exception of the Quality of Life Index (QLI) that included about 15 questions on satisfaction and importance of, for example, Peace of mind, Faith in God, and Achievement of personal goals.

#### Aspects not covered in the ICF or aspects that are not defined

Single PROMs asked individual questions about aspects that are not covered by the ICF or are not defined, including “Deteriorated life situation” (asked in the Arrhythmia-specific Questionnaire in Tachycardia and Arrhythmia (ASTA)) and “Staying at the hospital” (asked in the Minnesota Living with Heart Failure Questionnaire (MLHF)), or are time-related aspects like “Duration of treatment’ (asked in the QLAF), “Cutting down on the things that usually need to be done” (asked in the PPAQ), and “Limitations in daily life/day-to-day activities” (asked in 8 PROMs).

## Discussion

With this work we provide an overview of existing heart disease-specific PROMs and compare the content of these PROMs using the ICF as a framework. Thirty-four disease-specific PROMs for use in heart diseases were identified (additionally, there were short forms of two of the included PROMs), and a total of 147 different ICF categories in the WHO’s bio-psycho-social framework were identified in the PROMs. Most items concerned experienced problems of *Body functions*, but less often asked about the impact of these symptoms on patients’ daily activities. Moreover, this study showed that most PROMs were specifically developed for heart failure, and no PROMs were identified for valve diseases or for congenital heart disease.

With the increasing interest in using PROMs, there is a need for healthcare professionals and/or researchers to choose the most appropriate PROM. It is thus of importance to carefully consider the content of the PROM before its use in order to ensure that a given PROM covers important aspects of interest for care management of individual patients and for quality improvement [14, 15, 62, 63]. The present review and content comparison revealed that, for example, for heart disease in general the most comprehensive PROM was the QLI-cardiac version that focuses on problems in major life areas (e.g. employment and education) as well as support and relationships with family and friends. If focus should be on activities of daily living, e.g. washing, dressing, walking, and climbing stairs, the DASI might be more appropriate to choose, whereas the Multidimensional Index of Life Quality (MILQ) might be more appropriate if the focus is on mental functions like energy level, motivation, and mood.

In cardiac arrhythmia, the most comprehensive disease-specific instrument was the PPAQ with a main focus on problems within mental, sensory, and cardiovascular functions but also with a broader view on problems regarding mobility, household tasks, major life areas, interactions, and recreation. For heart failure, the MSAS-HF covers the widest range of different aspects but with a main focus on problems within mental, sensory, and cardiovascular functions and only one item about *Activities & Participation* (“Focusing attention”). If the focus should be on activities of daily living, recreation and leisure activities, medication, and support, then the Left Ventricular Dysfunction Questionnaire (LVD-36) might be more appropriate. For ischemic heart disease, the Summary Index for the Assessment of Quality of Life in Angina Pectoris covers most different aspects. The core of this instrument is a broad spectrum of mental functions, e.g. mood, energy level, motivation, emotions, sleep, attention, and thoughts, but it also addresses *Activities & Participation*, e.g. walking and climbing stairs, doing housework, and aspects related to work, recreation, and leisure.

The present study is the first content comparison of heart disease-specific PROMs. It showed that different PROMs focus on different aspects of QoL and that questions on *Body functions* were the most frequently asked and were included in all 34 PROMs. Obviously, biomedical issues like heart functions, the sensations with cardiovascular functions, emotions, and energy level were dominant in the PROMs. However, our results have identified other issues that were only addressed in a few PROMs, for example, recreation and leisure activities, climbing stairs, intimate and family relationships, medication tolerance, and support from family. Those issues are as well included in the ICF Core Set (ICF-CS) for ischemic heart disease [64]. The latter can be seen as an international standard of ICF categories important for patients with heart diseases that is based on a consensus and decision-making process involving international experts from diverse disciplines representing the six WHO world regions. Prior to the consensus process, the experts were provided with evidence from the clinical (multicenter study), health professionals’ (expert survey), researchers’ (literature review) perspective and the perspective of patients with the cardiological conditions (qualitative study) [65]. With this convenient evidence, it is argued that the identified issues are worth addressing in clinical counseling and should be included in PROMs in order to capture patients’ experiences of their health status from a bio-psycho-social perspective.

There are some limitations of the present study that should be mentioned. First, only PROMs measuring health, health-related states, QoL, and symptoms and available in English and validated for use in adults were included. This means that our results are valid for the adult patient with heart disease and not focusing on any single symptom such as fatigue or locus of control. Second, the literature search was carried out in PubMed only and the search was limited to the title. PubMed is the largest database for medical research and therefore judged as most relevant. The strategy to limit the search to the title was conducted to delimit the results since some of the search terms are very broad and resulted initially in a very large number of articles of no relevance without this restriction. The search strategy and terms were also discussed with a librarian with special competence in medical science and literature searches. Additionally, the CESAR expert group, consisting of more than 20 researchers within cardiovascular disease, confirmed the result and cud only present additionally four PROMs not identified in the literature search. Third, the linking of the PROMs was performed by two researchers very familiar with the ICF and the application of the ICF linking rules. Other researchers might have come up with slightly different linking results, but this is not likely to have significantly altered the conclusions drawn here.

## Conclusions

This scoping review and mapping exercises give an overview of several PROMs available for patients with different types of heart diseases and provide a detailed comparison of their content. These findings can help clinicians and researchers to identify PROMs of high relevance depending on which aspects they want to focus on. A great majority of the PROMs have been developed for a specific heart disease (mainly for heart failure), and none exist for valve diseases or congenital heart diseases. However, many patients suffer from more than one heart disease at the same time, and this stresses the importance of generic PROMs to be used for all different types of heart diseases. This study identified 10 *Body functions*, 13 aspects of *Activities & Participation*, and 2 aspects of *Environmental factors* that should be included in a generic PROM for patients with different types of heart diseases in order to capture patients’ experiences of their health status from a bio-psycho-social perspective. The results of the present study might be a good starting point for further development of PROMs for patients with heart diseases.

**Table:** 

What’s new	
• There are 5 PROMs for heart diseases in general, 6 disease-specific PROMs for cardiac arrhythmia, 14 PROMs for heart failure, and 9 PROMs for ischemic heart disease.	
• There is a lack of disease-specific PROMs for valve disease and congenital heart disease.	
• From a bio-psycho-social perspective, all PROMs included aspects of *Body functions*, but *Activities & Participation* and *Environmental factors* were less often included (missing in 6 and 18 PROMs, respectively).	
• The most common specific aspects were about individuals’ emotions and mood, individuals’ energy level and motivation, and individuals’ sensations with cardiovascular functions.	
• Less common specific aspects were about medication tolerance, support of family, doing housework, employment, sleep, and sexual relationship, and these were only included in few PROMs.	
• There is a need for further development of heart disease-specific PROMs that can support person-centered care.	

## Supplementary information

**Additional file 1.** Overview of the Pubmed search strings that were used to identify specific PROMs for heart diseases in general, cardiac arrhythmia, heart failure, ischemic heart disease, valve disease and congenital heart disease. The literature search was conducted in January 2018.

**Additional file 2. **Overview of different aspects measured by each PROMs (*n* = 34) linked to ICF catgories from the component ‘*Body Functions*’ stratified by heart diseases.

**Additional file 3. **Overview of different aspects measured by each PROMs (*n* = 34) linked to ICF catgories from the component ‘*Activities and Participation*’ stratified by heart diseases.

**Additional file 4. **Overview of different aspects measured by each PROMs (*n* = 34) linked to ICF catgories from the component ‘*Environmental factors’* stratified by heart diseases.

## Data Availability

This article is entirely based on data and materials that have been published, are publicly available (thus, accessible to any interested researcher), and appear in the references list.

## Abbreviations

ICF: International Classification of Functioning, Disability and Health
PROM: Patient-reported outcome measure
QoL: Quality of life
WHO: World Health Organization
