# Supplementary material for: A scoping review and mapping exercise comparing the content of patient-reported outcome measures (PROMs) across heart disease-specific scales
Source: J Patient Rep Outcomes. 2020 Jan 23;4:7. doi: 10.1186/s41687-019-0165-7 (PMC6977790; doi:10.1186/s41687-019-0165-7)
Supplement: Supplementary file 1 — Additional file 1. Overview of the Pubmed search strings that were used to identify specific PROMs for heart diseases in general, cardiac arrhythmia, heart failure, ischemic heart disease, valve disease and congenital heart disease. The literature search was conducted in January 2018. [file 41687_2019_165_MOESM1_ESM.docx]

Additional file 1. Overview of the Pubmed search strings that were used to identify specific PROMs for heart diseases in general, cardiac arrhythmia, heart failure, ischemic heart disease, valve disease and congenital heart disease. The literature search was conducted in January 2018.

| Heart diseases in general | ((heart disease*[Title]) AND (questionnaire*[Title])) OR ((heart disease*[Title]) AND (valid*[Title]) AND (scale*[Title])) OR ((heart disease*[Title]) AND (valid*[Title]) AND (index*[Title])) OR ((heart disease*[Title]) AND (valid*[Title]) AND (instrument*[Title])) OR ((heart disease*[Title]) AND (valid*[Title]) AND (questionnaire*[Title])) OR ((heart disease*[Title]) AND (develop*[Title]) AND (scale*[Title])) OR ((heart disease*[Title]) AND (develop*[Title]) AND (index*[Title])) OR ((heart disease*[Title]) AND (develop*[Title]) AND (instrument*[Title])) |
| --- | --- |
|  |  |
|  | ((cardiac disease*[Title]) AND (questionnaire*[Title])) OR ((cardiac disease*[Title]) AND (valid*[Title]) AND (scale*[Title])) OR ((cardiac disease*[Title]) AND (valid*[Title]) AND (index*[Title])) OR ((cardiac disease*[Title]) AND (valid*[Title]) AND (instrument*[Title])) OR ((cardiac disease*[Title]) AND (valid*[Title]) AND (questionnaire*[Title])) OR ((cardiac disease*[Title]) AND (develop*[Title]) AND (scale*[Title])) OR ((cardiac disease*[Title]) AND (develop*[Title]) AND (index*[Title])) OR ((cardiac disease*[Title]) AND (develop*[Title]) AND (instrument*[Title])) |
|  |  |
| Cardiac arrhythmia | ((arrhythmia[Title]) AND (questionnaire*[Title])) OR ((arrhythmia[Title]) AND (valid*[Title]) AND (scale*[Title])) OR ((arrhythmia[Title]) AND (valid*[Title]) AND (index*[Title])) OR ((arrhythmia[Title]) AND (valid*[Title]) AND (instrument*[Title])) OR ((arrhythmia[Title]) AND (valid*[Title]) AND (questionnaire*[Title])) OR ((arrhythmia[Title]) AND (develop*[Title]) AND (scale*[Title])) OR ((arrhythmia[Title]) AND (develop*[Title]) AND (index*[Title])) OR ((arrhythmia[Title]) AND (develop*[Title]) AND (instrument*[Title])) |
|  |  |
|  | ((atrial[Title]) AND (questionnaire*[Title])) OR ((atrial[Title]) AND (valid*[Title]) AND (scale*[Title])) OR ((atrial[Title]) AND (valid*[Title]) AND (index*[Title])) OR ((atrial[Title]) AND (valid*[Title]) AND (instrument*[Title])) OR ((atrial[Title]) AND (valid*[Title]) AND (questionnaire*[Title])) OR ((atrial[Title]) AND (develop*[Title]) AND (scale*[Title])) OR ((atrial[Title]) AND (develop*[Title]) AND (index*[Title])) OR ((atrial[Title]) AND (develop*[Title]) AND (instrument*[Title])) |
|  |  |
| Heart failure | ((heart failure*[Title]) AND (questionnaire*[Title])) OR ((heart failure*[Title]) AND (valid*[Title]) AND (scale*[Title])) OR ((heart failure*[Title]) AND (valid*[Title]) AND (index*[Title])) OR ((heart failure*[Title]) AND (valid*[Title]) AND (instrument*[Title])) OR ((heart failure*[Title]) AND (valid*[Title]) AND (questionnaire*[Title])) OR ((heart failure*[Title]) AND (develop*[Title]) AND (scale*[Title])) OR ((heart failure*[Title]) AND (develop*[Title]) AND (index*[Title])) OR ((heart failure*[Title]) AND (develop*[Title]) AND (instrument*[Title])) |
|  |  |
|  |  |
| Ischemic heart disease | ((ischemic heart disease*[Title]) AND (questionnaire*[Title])) OR ((ischemic heart disease*[Title]) AND (valid*[Title]) AND (scale*[Title])) OR ((ischemic heart disease*[Title]) AND (valid*[Title]) AND (index*[Title])) OR ((ischemic heart disease*[Title]) AND (valid*[Title]) AND (instrument*[Title])) OR ((ischemic heart disease*[Title]) AND (valid*[Title]) AND (questionnaire*[Title])) OR ((ischemic heart disease*[Title]) AND (develop*[Title]) AND (scale*[Title])) OR ((ischemic heart disease*[Title]) AND (develop*[Title]) AND (index*[Title])) OR ((ischemic heart disease*[Title]) AND (develop*[Title]) AND (instrument*[Title])) |
|  |  |
|  | ((angina*[Title]) AND (questionnaire*[Title])) OR ((angina*[Title]) AND (valid*[Title]) AND (scale*[Title])) OR ((angina*[Title]) AND (valid*[Title]) AND (index*[Title])) OR ((angina*[Title]) AND (valid*[Title]) AND (instrument*[Title])) OR ((angina*[Title]) AND (valid*[Title]) AND (questionnaire*[Title])) OR ((angina*[Title]) AND (develop*[Title]) AND (scale*[Title])) OR ((angina*[Title]) AND (develop*[Title]) AND (index*[Title])) OR ((angina*[Title]) AND (develop*[Title]) AND (instrument*[Title])) |
|  |  |
|  | ((myoc*[Title]) AND (questionnaire*[Title])) OR ((myoc*[Title]) AND (valid*[Title]) AND (scale*[Title])) OR ((myoc*[Title]) AND (valid*[Title]) AND (index*[Title])) OR ((myoc*[Title]) AND (valid*[Title]) AND (instrument*[Title])) OR ((myoc*[Title]) AND (valid*[Title]) AND (questionnaire*[Title])) OR ((myoc*[Title]) AND (develop*[Title]) AND (scale*[Title])) OR ((myoc*[Title]) AND (develop*[Title]) AND (index*[Title])) OR ((myoc*[Title]) AND (develop*[Title]) AND (instrument*[Title])) |
|  |  |
| Valve disease | ((valve heart disease*[Title]) AND (questionnaire*[Title])) OR ((valve heart disease*[Title]) AND (valid*[Title]) AND (scale*[Title])) OR ((valve heart disease*[Title]) AND (valid*[Title]) AND (index*[Title])) OR ((valve heart disease*[Title]) AND (valid*[Title]) AND (instrument*[Title])) OR ((valve heart disease*[Title]) AND (valid*[Title]) AND (questionnaire*[Title])) OR ((valve heart disease*[Title]) AND (develop*[Title]) AND (scale*[Title])) OR ((valve heart disease*[Title]) AND (develop*[Title]) AND (index*[Title])) OR ((valve heart disease*[Title]) AND (develop*[Title]) AND (instrument*[Title])) |
|  |  |
| Congenital heart disease | ((congenital heart disease*[Title]) AND (congenital heart disease*[Title])) OR ((congenital heart disease*[Title]) AND (valid*[Title]) AND (scale*[Title])) OR ((congenital heart disease*[Title]) AND (valid*[Title]) AND (index*[Title])) OR ((congenital heart disease*[Title]) AND (valid*[Title]) AND (instrument*[Title])) OR ((congenital heart disease*[Title]) AND (valid*[Title]) AND (questionnaire*[Title])) OR ((congenital heart disease*[Title]) AND (develop*[Title]) AND (scale*[Title])) OR ((congenital heart disease*[Title]) AND (develop*[Title]) AND (index*[Title])) OR ((congenital heart disease*[Title]) AND (develop*[Title]) AND (instrument*[Title])) |
