# Supplementary material for: A scoping review and mapping exercise comparing the content of patient-reported outcome measures (PROMs) across heart disease-specific scales
Source: J Patient Rep Outcomes. 2020 Jan 23;4:7. doi: 10.1186/s41687-019-0165-7 (PMC6977790; doi:10.1186/s41687-019-0165-7)
Supplement: Supplementary file 2 — Additional file 2. Overview of different aspects measured by each PROMs (n = 34) linked to ICF catgories from the component ‘Body Functions’ stratified by heart diseases. [file 41687_2019_165_MOESM2_ESM.docx]

| Additional file 2. Overview of different aspects measured by each PROMs (n=34)* linked to ICF catgories from the component 'Body Functions' stratified by heart diseases | | | | | | | | | | | | | | | | | | | | | | | | |  |  |  |  |  |  |  |  |  |  |  |  |
| --- | --- | --- | --- | --- | --- | --- | --- | --- | --- | --- | --- | --- | --- | --- | --- | --- | --- | --- | --- | --- | --- | --- | --- | --- | --- | --- | --- | --- | --- | --- | --- | --- | --- | --- | --- | --- |
|  |  |  |  |  |  |  |  |  |  |  |  |  |  |  |  |  |  |  |  |  |  |  |  |  |  |  |  |  |  |  |  |  |  |  |  |  |
| ICF categories | | Heart diseases in general | | | | | Cardiac arrythmia | | | | | |  |  |  |  | Heart failure | | | | | | | | |  |  | Ischemic heart disease | | | | | | | |  |
| ICF code | ICF title | DASI | HeartQoL | MILQ | QLI - cardic version | QLICD-CHD | AF-QoL-40 | ASTA | AF6 | AFEQT | PPAQ | QLAF | CHPchf | CaReQoL CHF | CHQ | CHD-TAAQOL | FEW16 | IDCV | HFSAS | KCCQ | LVD-36 | MSAS-HF | MLHF | QLQ-SHF | SSQ-HF | TCM inquiry | ALWQ | APQOL | CLASP | MIDAS | QLI | QLMI-2 | SAQ | Summary Index for the Assessment of Quality of Life in Angina Pectoris | WHO Rose Angina Questionnaire | **n** |
| b1 | *Mental Functions* | 0 | 3 | 5 | 2 | 3 | 8 | 6 | 2 | 3 | 5 | 0 | 3 | 5 | 2 | 1 | 3 | 4 | 1 | 2 | 5 | 6 | 5 | 6 | 2 | 5 | 2 | 5 | 3 | 6 | 4 | 6 | 1 | 14 | 1 | 37 |
| b110 | Consciousness functions |  |  |  |  |  |  | x |  |  | x |  |  |  |  |  |  |  |  |  |  |  |  |  |  |  |  |  |  |  |  |  |  |  |  | 2 |
| b1100 | State of consciousness |  |  |  |  |  |  |  |  | x |  |  |  |  |  |  |  |  |  |  |  |  |  |  |  |  |  |  |  |  |  |  |  |  |  | 1 |
| b126 | Temperament and personality functions |  |  |  |  |  |  |  |  |  |  |  |  |  |  |  |  |  |  |  |  |  |  |  |  |  |  |  |  | x |  | x |  | x |  | 3 |
| b1263 | Psychic stability |  | x | x |  |  | x | x |  |  |  |  |  | x |  |  |  | x |  |  | x | x |  | x |  | x | x | x |  | x | x | x |  | x |  | 16 |
| b1265 | Optimism |  |  | x |  |  | x |  |  |  |  |  |  | x |  |  |  |  |  |  |  |  |  | x |  |  |  | x |  |  |  | x |  | x |  | 7 |
| b1266HF | Confidence |  |  |  |  | x | x |  |  |  |  |  |  |  |  |  | x |  |  |  | x |  |  | x |  |  |  |  |  | x |  | x |  | x |  | 8 |
| b1268 | Temperament and personality functions, other specified |  |  |  |  |  | x |  |  |  |  |  |  |  |  |  |  |  |  |  |  |  |  |  |  |  |  |  |  |  |  |  |  |  |  | 1 |
| b130 | Energy and drive functions |  |  |  |  |  |  |  |  |  |  |  |  |  |  |  |  |  |  |  |  |  |  |  |  |  |  | x |  |  |  |  |  |  |  | 1 |
| b1300 | Energy level |  | x | x | x |  | x | x | x | x | x |  | x | x |  |  | x | x | x | x | x | x | x | x | x | x |  | x | x | x | x | x |  | x |  | 26 |
| b1301 | Motivation |  |  |  |  |  | x |  |  |  |  |  |  |  |  |  |  |  |  |  |  |  |  | x |  | x |  |  |  | x |  |  |  | x |  | 5 |
| b1302 | Appetite |  |  |  |  |  |  |  |  |  | x |  | x |  |  |  |  |  |  |  |  | x |  |  |  |  |  |  |  |  |  |  |  |  |  | 3 |
| b134 | Sleep functions |  |  |  |  |  |  | x |  |  | x |  |  | x | x |  | x | x |  |  |  | x |  |  | x |  |  |  |  |  |  |  |  |  |  | 8 |
| b1340 | Amount of sleep |  |  |  |  |  |  |  |  |  |  |  |  |  |  |  |  |  |  |  |  |  |  |  |  |  |  |  |  |  | x |  |  |  |  | 1 |
| b1341 | Onset of sleep |  |  |  |  |  |  |  |  |  |  |  |  |  |  |  |  |  |  |  |  |  |  |  |  |  |  |  |  |  |  |  |  | x |  | 1 |
| b1342 | Maintenance of sleep |  |  |  |  |  | x |  |  |  |  |  |  |  |  |  |  |  |  |  | x |  |  |  |  |  |  |  | x |  |  |  |  | x |  | 4 |
| b140 | Attention functions |  |  |  |  |  |  | x |  |  |  |  |  |  |  |  |  |  |  |  |  |  | x |  |  |  |  |  |  |  |  |  |  |  |  | 2 |
| b144 | Memory functions |  |  | x |  |  |  |  |  |  |  |  |  |  |  |  |  |  |  |  |  |  | x |  |  | x |  |  |  |  |  |  |  | x |  | 4 |
| b152 | Emotional functions |  | x | x | x | x | x | x | x | x | x |  | x | x | x | x |  | x |  | x | x | x | x | x |  | x | x | x | x | x | x | x | x | x | x | 29 |
| b1521 | Regulation of emotion |  |  |  |  | x |  |  |  |  |  |  |  |  |  |  |  |  |  |  |  |  |  |  |  |  |  |  |  |  |  |  |  | x |  | 2 |
| b1563 | Gustatory perception |  |  |  |  |  |  |  |  |  |  |  |  |  |  |  |  |  |  |  |  | x |  |  |  |  |  |  |  |  |  |  |  |  |  | 1 |
| b160 | Thought functions |  |  |  |  |  |  |  |  |  |  |  |  |  |  |  |  |  |  |  |  |  |  |  |  |  |  |  |  |  |  |  |  | x |  | 1 |
| b1603 | Control of thought |  |  |  |  |  |  |  |  |  |  |  |  |  |  |  |  |  |  |  |  |  |  |  |  |  |  |  |  |  |  |  |  | x |  | 1 |
| b167 | Mental functions of language |  |  |  |  |  |  |  |  |  |  |  |  |  |  |  |  |  |  |  |  |  |  |  |  |  |  |  |  |  |  |  |  | x |  | 1 |
| b2 | *Sensory functions and pain* | 0 | 0 | 1 | 1 | 4 | 0 | 2 | 0 | 1 | 5 | 2 | 0 | 0 | 0 | 1 | 0 | 1 | 0 | 0 | 0 | 4 | 0 | 1 | 3 | 4 | 0 | 1 | 0 | 3 | 1 | 3 | 1 | 2 | 2 | 43 |
| b2102 | Quality of vision |  |  |  |  |  |  |  |  |  | x |  |  |  |  |  |  |  |  |  |  |  |  |  |  |  |  |  |  |  |  |  |  |  |  | 1 |
| b21023 | Visual picture quality |  |  |  |  |  |  |  |  |  |  |  |  |  |  |  |  |  |  |  |  |  |  |  |  | x |  |  |  |  |  |  |  |  |  | 1 |
| b2351 | Vestibular function of balance |  |  |  |  |  |  |  |  |  |  |  |  |  |  |  |  |  |  |  |  |  |  |  | x |  |  |  |  |  |  |  |  |  |  | 1 |
| b2401 | Dizziness |  |  |  |  |  |  | x |  | x | x | x |  |  |  | x |  | x |  |  |  | x |  |  | x | x |  |  |  |  |  | x |  |  |  | 10 |
| b2403 | Nausea associated with dizziness or vertigo |  |  |  |  |  |  |  |  |  | x |  |  |  |  |  |  |  |  |  |  |  |  |  |  |  |  |  |  |  |  |  |  |  |  | 1 |
| b265 | Touch function |  |  |  |  |  |  |  |  |  |  |  |  |  |  |  |  |  |  |  |  | x |  |  |  |  |  |  |  |  |  |  |  |  |  | 1 |
| b270 | Sensory functions related to temperature and other stimuli |  |  |  |  |  |  |  |  |  |  |  |  |  |  |  |  |  |  |  |  |  |  |  |  |  |  |  |  | x |  |  |  |  |  | 1 |
| b2700 | Sensitivity to temperature |  |  |  |  |  |  |  |  |  |  |  |  |  |  |  |  |  |  |  |  |  |  |  |  | x |  |  |  |  |  |  |  |  |  | 1 |
| b280 | Sensation of pain |  |  | x |  | x |  |  |  |  |  |  |  |  |  |  |  |  |  |  |  | x |  |  |  |  |  |  |  | x |  |  |  | x |  | 5 |
| b2801 | Pain in body part |  |  |  |  |  |  |  |  |  |  |  |  |  |  |  |  |  |  |  |  |  |  |  |  |  |  |  |  |  | x |  |  |  |  | 1 |
| b28010 | Pain in head and neck |  |  |  |  |  |  |  |  |  | x |  |  |  |  |  |  |  |  |  |  |  |  |  |  |  |  |  |  |  |  |  |  |  |  | 1 |
| b28011 | Pain in chest |  |  |  | x | x |  | x |  |  | x | x |  |  |  |  |  |  |  |  |  | x |  | x | x | x |  | x |  | x |  | x | x | x | **x** ^§^ | 14 |
| b28012 | Pain in stomach or abdomen |  |  |  |  | x |  |  |  |  |  |  |  |  |  |  |  |  |  |  |  |  |  |  |  |  |  |  |  |  |  |  |  |  |  | 1 |
| b28014 | Pain in upper limb |  |  |  |  | x |  |  |  |  |  |  |  |  |  |  |  |  |  |  |  |  |  |  |  |  |  |  |  |  |  |  |  |  |  | 1 |
| b28015 | Pain in lower limb |  |  |  |  |  |  |  |  |  |  |  |  |  |  |  |  |  |  |  |  |  |  |  |  |  |  |  |  |  |  | x |  |  | x | 2 |
| b4 | *Functions of the cardiovascular, haematological, immunological and respiratory systems* | 0 | 1 | 0 | 1 | 1 | 3 | 4 | 3 | 3 | 5 | 1 | 5 | 0 | 0 | 3 | 0 | 1 | 4 | 3 | 3 | 4 | 3 | 2 | 3 | 3 | 1 | 2 | 3 | 1 | 0 | 3 | 1 | 2 | 1 | 70 |
| b410 | Heart functions |  |  |  |  |  |  |  |  |  | x |  |  |  |  |  |  |  |  |  |  |  |  |  |  |  |  |  |  |  |  | x |  |  |  | 2 |
| b4100 | Heart rate |  |  |  |  |  | x |  |  |  | x |  |  |  |  |  |  |  |  |  |  |  |  |  |  |  |  |  |  |  |  |  |  |  |  | 2 |
| b4101 | Heart rhythm |  |  |  |  |  |  | x |  |  | x |  |  |  |  |  |  |  |  |  |  |  |  |  |  |  |  |  |  |  |  |  |  |  |  | 2 |
| b4352 | Functions of lymphatic vessels |  |  |  |  |  |  |  |  |  |  |  |  |  |  |  |  |  | x | x | x | x |  |  | x |  |  |  |  |  |  |  |  |  |  | 5 |
| b440 | Respiration functions |  |  |  |  |  |  |  |  |  |  |  |  |  |  |  |  |  | x |  |  | x |  |  |  |  |  |  |  |  |  |  |  |  | x | 3 |
| b450 | Additional respiratory functions |  |  |  |  |  |  |  |  |  |  |  |  |  |  |  |  |  |  |  |  | x |  |  |  |  |  |  |  |  |  |  |  |  |  | 1 |
| b455 | Exercise tolerance functions |  |  |  |  |  |  | x | x |  |  |  |  |  |  |  |  |  |  |  |  |  |  |  |  |  | x |  |  |  |  |  |  | x |  | 4 |
| b4551 | Aerobic capacity |  |  |  |  |  |  |  |  | x |  |  | x |  |  |  |  |  | x |  | x |  |  |  |  | x |  |  |  |  |  |  |  |  |  | 5 |
| b4552 | Fatiguability |  | x |  |  |  | x | x | x | x | x |  | x |  |  |  |  |  |  | x |  |  | x | x | x | x |  | x | x |  |  |  |  | x |  | 15 |
| b4558 | Exercise tolerance functions, other specified |  |  |  |  |  |  |  |  |  |  |  | x |  |  | x |  |  |  |  | x |  |  |  |  |  |  |  |  |  |  | x |  |  |  | 4 |
| b460 | Sensations associated with cardiovascular and respiratory functions |  |  |  | x | x | x | x | x | x | x | x | x |  |  | x |  | x | x | x |  | x | x | x | x | x |  | x | x | x |  | x | x |  |  | 23 |
| b498 | Functions of the cardiovascular, haematological, immunological and respiratory systems, other specified |  |  |  |  |  |  |  |  |  |  |  | x |  |  | x |  |  |  |  |  |  | x |  |  |  |  |  | x |  |  |  |  |  |  | 4 |
| b5 | *Functions of the digestive, metabolic and endocrine systems* | 0 | 0 | 0 | 0 | 0 | 0 | 0 | 0 | 0 | 2 | 0 | 0 | 0 | 0 | 0 | 0 | 0 | 2 | 0 | 2 | 7 | 0 | 0 | 0 | 0 | 0 | 0 | 0 | 1 | 1 | 0 | 0 | 0 | 0 | 15 |
| b5104 | Salivation |  |  |  |  |  |  |  |  |  |  |  |  |  |  |  |  |  |  |  |  | x |  |  |  |  |  |  |  |  |  |  |  |  |  | 1 |
| b5106 | Regurgitation and vomiting |  |  |  |  |  |  |  |  |  |  |  |  |  |  |  |  |  |  |  |  | x |  |  |  |  |  |  |  |  |  |  |  |  |  | 1 |
| b5251 | Faecal consistency |  |  |  |  |  |  |  |  |  |  |  |  |  |  |  |  |  |  |  |  | x |  |  |  |  |  |  |  |  |  |  |  |  |  | 1 |
| b5252 | Frequency of defecation |  |  |  |  |  |  |  |  |  |  |  |  |  |  |  |  |  |  |  |  | x |  |  |  |  |  |  |  |  |  |  |  |  |  | 1 |
| b530 | Weight maintenance functions |  |  |  |  |  |  |  |  |  |  |  |  |  |  |  |  |  | x |  |  | x |  |  |  |  |  |  |  | x | x |  |  |  |  | 4 |
| b5350 | Sensation of nausea |  |  |  |  |  |  |  |  |  | x |  |  |  |  |  |  |  |  |  | x | x |  |  |  |  |  |  |  |  |  |  |  |  |  | 3 |
| b5351 | Feeling bloated |  |  |  |  |  |  |  |  |  |  |  |  |  |  |  |  |  | x |  |  | x |  |  |  |  |  |  |  |  |  |  |  |  |  | 2 |
| b550 | Thermoregulatory functions |  |  |  |  |  |  |  |  |  | x |  |  |  |  |  |  |  |  |  | x |  |  |  |  |  |  |  |  |  |  |  |  |  |  | 2 |
| b6 | *Genitourinary and reproductive functions* | 0 | 0 | 0 | 0 | 1 | 1 | 1 | 0 | 0 | 1 | 0 | 0 | 0 | 0 | 1 | 0 | 0 | 0 | 0 | 0 | 2 | 0 | 0 | 0 | 0 | 0 | 0 | 1 | 0 | 1 | 0 | 0 | 0 | 0 | 9 |
| b620 | Urination functions |  |  |  |  |  |  |  |  |  | x |  |  |  |  | x |  |  |  |  |  | x |  |  |  |  |  |  |  |  |  |  |  |  |  | 3 |
| b640 | Sexual functions |  |  |  |  | x | x | x |  |  |  |  |  |  |  |  |  |  |  |  |  | x |  |  |  |  |  |  | x |  | x |  |  |  |  | 6 |
| b7 | *Neuromusculoskeletal and movement-related functions* | 0 | 0 | 0 | 0 | 0 | 0 | 0 | 0 | 0 | 0 | 0 | 0 | 0 | 0 | 0 | 0 | 0 | 0 | 0 | 1 | 0 | 0 | 0 | 0 | 1 | 0 | 0 | 0 | 0 | 0 | 0 | 0 | 0 | 0 | 2 |
| b730 | Muscle power functions |  |  |  |  |  |  |  |  |  |  |  |  |  |  |  |  |  |  |  | x |  |  |  |  |  |  |  |  |  |  |  |  |  |  | 1 |
| b780 | Sensations related to muscle and movement functions |  |  |  |  |  |  |  |  |  |  |  |  |  |  |  |  |  |  |  |  |  |  |  |  | x |  |  |  |  |  |  |  |  |  | 1 |
| b8 | *Functions of the skin and related structures* | 0 | 0 | 0 | 0 | 0 | 0 | 1 | 0 | 0 | 1 | 0 | 0 | 0 | 0 | 0 | 0 | 0 | 0 | 0 | 0 | 2 | 0 | 0 | 0 | 0 | 0 | 0 | 0 | 0 | 0 | 0 | 0 | 0 | 0 | 4 |
| b830 | Other functions of the skin |  |  |  |  |  |  | x |  |  | x |  |  |  |  |  |  |  |  |  |  | x |  |  |  |  |  |  |  |  |  |  |  |  |  | 3 |
| b840 | Sensation related to the skin |  |  |  |  |  |  |  |  |  |  |  |  |  |  |  |  |  |  |  |  | x |  |  |  |  |  |  |  |  |  |  |  |  |  | 1 |
| % (x) |  | 0 | 7 | 10 | 7 | 15 | 20 | 23 | 8 | 11 | 31 | 5 | 13 | 8 | 3 | 10 | 5 | 10 | 11 | 8 | 18 | 41 | 11 | 15 | 13 | 21 | 5 | 13 | 11 | 18 | 11 | 20 | 5 | 30 | 5 |  |
| n (x) | 61 | 0 | 4 | 6 | 4 | 9 | 12 | 14 | 5 | 7 | 19 | 3 | 8 | 5 | 2 | 6 | 3 | 6 | 7 | 5 | 11 | 25 | 7 | 9 | 8 | 13 | 3 | 8 | 7 | 11 | 7 | 12 | 3 | 18 | 3 |  |
