# Supplementary material for: A scoping review and mapping exercise comparing the content of patient-reported outcome measures (PROMs) across heart disease-specific scales
Source: J Patient Rep Outcomes. 2020 Jan 23;4:7. doi: 10.1186/s41687-019-0165-7 (PMC6977790; doi:10.1186/s41687-019-0165-7)
Supplement: Supplementary file 3 — Additional file 3. Overview of different aspects measured by each PROMs (n = 34) linked to ICF catgories from the component ‘Activities and Participation’ stratified by heart diseases. [file 41687_2019_165_MOESM3_ESM.docx]

| Additional file 3. Overview of different aspects measured by each PROMs (n=34)* linked to ICF catgories from the component 'Activities and Participation' stratified by heart diseases | | | | | | | | | | | | | | | | | | | | | | | | | | | | | |  |  |  |  |  |  |  |
| --- | --- | --- | --- | --- | --- | --- | --- | --- | --- | --- | --- | --- | --- | --- | --- | --- | --- | --- | --- | --- | --- | --- | --- | --- | --- | --- | --- | --- | --- | --- | --- | --- | --- | --- | --- | --- |
|  |  |  |  |  |  |  |  |  |  |  |  |  |  |  |  |  |  |  |  |  |  |  |  |  |  |  |  |  |  |  |  |  |  |  |  |  |
| ICF categories | | Heart diseases in general | | | | | Cardiac arrythmia | | | | | |  | Heart failure | | | | | | | | | | |  | | Ischemic heart disease | | | | | | | | | Total |
| ICF code | ICF title | DASI | HeartQoL | MILQ | QLI - cardic version | QLICD-CHD | AF-QoL-40 | ASTA | AF6 | AFEQT | PPAQ | QLAF | CHPchf | CaReQoL CHF | CHQ | CHD-TAAQOL | FEW16 | IDCV | HFSAS | KCCQ | LVD-36 | MSAS | MLHF | QLQ-SHF | SSQ-HF | TCM inquiry | ALWQ | APQOL | CLASP | MIDAS | QLI | QLMI-2 | SAQ | Summary Index for the Assessment of Quality of Life in Angina Pectoris | WHO Rose Angina questionnaire | **n** |
| *D1* | *Learning and applying knowledge* | 0 | 0 | 1 | 0 | 0 | 0 | 0 | 0 | 0 | 0 | 0 | 0 | 0 | 0 | 0 | 0 | 0 | 0 | 0 | 0 | 1 | 0 | 0 | 0 | 0 | 1 | 0 | 0 | 0 | 0 | 0 | 0 | 1 | 0 | 4 |
| d160 | Focusing attention |  |  |  |  |  |  |  |  |  |  |  |  |  |  |  |  |  |  |  |  | x |  |  |  |  | x |  |  |  |  |  |  | x |  | 3 |
| d177 | Making decisions |  |  | x |  |  |  |  |  |  |  |  |  |  |  |  |  |  |  |  |  |  |  |  |  |  |  |  |  |  |  |  |  |  |  | 1 |
| *d2* | *GENERAL TASKS AND DEMANDS* | 0 | 0 | 1 | 0 | 0 | 1 | 1 | 0 | 0 | 0 | 0 | 0 | 1 | 0 | 0 | 1 | 0 | 0 | 0 | 0 | 0 | 0 | 0 | 0 | 0 | 5 | 0 | 0 | 0 | 0 | 0 | 0 | 0 | 0 | 9 |
| d210 | Undertaking a single task |  |  |  |  |  |  |  |  |  |  |  |  |  |  |  |  |  |  |  |  |  |  |  |  |  | x |  |  |  |  |  |  |  |  | 1 |
| d230 | Carrying out daily routine |  |  |  |  |  |  | x |  |  |  |  |  | x |  |  |  |  |  |  |  |  |  |  |  |  | x |  |  |  |  |  |  |  |  | 3 |
| d2302 | Completing the daily rutine |  |  |  |  |  |  |  |  |  |  |  |  |  |  |  |  |  |  |  |  |  |  |  |  |  | x |  |  |  |  |  |  |  |  | 1 |
| d2303 | Managing own’s activity level |  |  |  |  |  |  |  |  |  |  |  |  |  |  |  | x |  |  |  |  |  |  |  |  |  | x |  |  |  |  |  |  |  |  | 2 |
| d240 | Handling stress and other psychological demands |  |  |  |  |  |  |  |  |  |  |  |  |  |  |  |  |  |  |  |  |  |  |  |  |  | x |  |  |  |  |  |  |  |  | 1 |
| *D3* | *Communication* | 0 | 0 | 0 | 0 | 0 | 0 | 0 | 0 | 0 | 0 | 0 | 0 | 0 | 1 | 0 | 0 | 0 | 0 | 0 | 0 | 0 | 0 | 0 | 0 | 0 | 0 | 0 | 0 | 0 | 0 | 0 | 0 | 0 | 0 | 1 |
| d330 | Speaking |  |  |  |  |  |  |  |  |  |  |  |  |  | x |  |  |  |  |  |  |  |  |  |  |  |  |  |  |  |  |  |  |  |  | 1 |
| *D4* | *Mobility* | 6 | 4 | 1 | 0 | 0 | 5 | 0 | 0 | 3 | 2 | 0 | 0 | 2 | 8 | 4 | 0 | 0 | 0 | 1 | 2 | 0 | 2 | 3 | 0 | 0 | 2 | 2 | 5 | 0 | 0 | 0 | 5 | 2 | 7 | 66 |
| d4105 | Bending |  |  |  |  |  |  |  |  |  |  |  |  |  | x |  |  |  |  |  |  |  |  |  |  |  |  |  | x |  |  |  |  |  | x | 3 |
| d4106 | Shifting the body's centre of gravity |  |  |  |  |  |  |  |  |  |  |  |  |  |  |  |  |  |  |  |  |  |  |  |  |  |  |  | x |  |  |  |  |  |  | 1 |
| d415 | Maintaining a body position |  |  |  |  |  |  |  |  |  |  |  |  |  |  |  |  |  |  |  |  |  |  |  |  |  |  |  |  |  |  |  |  |  | x | 1 |
| d4150 | Maintaining a lying position |  |  |  |  |  |  |  |  |  |  |  |  |  | x | x |  |  |  |  |  |  |  |  |  |  |  |  | x |  |  |  |  |  |  | 3 |
| d4154 | Maintaining a standing position |  |  |  |  |  |  |  |  |  |  |  |  |  |  |  |  |  |  |  |  |  |  |  |  |  |  |  |  |  |  |  |  |  | x | 1 |
| d430 | Lifting and carrying objects | **x** ^§^ |  | x |  |  |  |  |  | x |  |  |  |  | x |  |  |  |  |  |  |  |  |  |  |  | x |  |  |  |  |  | x |  |  | 5 |
| d4300 | Lifting |  | x |  |  |  |  |  |  |  |  |  |  |  |  |  |  |  |  |  |  |  |  |  |  |  |  |  |  |  |  |  | x |  |  | 2 |
| d4458 | Hand and arm use, other specified |  |  |  |  |  |  |  |  |  |  |  |  |  | x |  |  |  |  |  |  |  |  |  |  |  |  |  |  |  |  |  |  |  |  | 1 |
| d450 | Walking |  |  |  |  |  |  |  |  |  |  |  |  | x | x |  |  |  |  |  |  |  | x |  |  |  |  |  |  |  |  |  |  |  | **x** | 4 |
| d4500 | Walking short distances |  |  |  |  |  |  |  |  |  |  |  |  |  |  | x |  |  |  |  |  |  |  |  |  |  |  |  |  |  |  |  |  |  |  | 1 |
| d4501 | Walking long distances | **x** |  |  |  |  | x |  |  |  |  |  |  |  |  | x |  |  |  |  |  |  |  |  |  |  |  | x |  |  |  |  |  |  |  | 4 |
| d4508 | Walking, other specified |  | x |  |  |  | x |  |  | x |  |  |  |  | x |  |  |  |  |  |  |  |  |  |  |  |  |  |  |  |  |  |  |  | x | 5 |
| d455 | Moving around |  |  |  |  |  |  |  |  |  |  |  |  |  |  |  |  |  |  |  | x |  | x |  |  |  |  |  |  |  |  |  |  |  |  | 2 |
| d4551 | Climbing (e.g climbing stairs) | **x** | x |  |  |  | x |  |  | x |  |  |  |  | x | x |  |  |  |  |  |  |  | x |  |  |  |  | x |  |  |  | x | x | **x** | 11 |
| d4552 | Running | **x** |  |  |  |  | x |  |  |  |  |  |  |  | x |  |  |  |  | x | x |  |  |  |  |  |  | x |  |  |  |  | x | x | **x** | 9 |
| d460 | Moving around in different locations |  | x |  |  |  |  |  |  |  |  |  |  |  |  |  |  |  |  |  |  |  |  |  |  |  |  |  |  |  |  |  |  |  |  | 1 |
| d4600 | Moving around within the home | **x** |  |  |  |  |  |  |  |  |  |  |  |  |  |  |  |  |  |  |  |  |  | x |  |  |  |  |  |  |  |  |  |  |  | 2 |
| d4602 | Moving around outside the home and other buildings | **x** |  |  |  |  |  |  |  |  |  |  |  |  |  |  |  |  |  |  |  |  |  | x |  |  | x |  | x |  |  |  |  |  |  | 4 |
| d4608 | Moving around in different locations, other specified |  |  |  |  |  |  |  |  |  |  |  |  |  |  |  |  |  |  |  |  |  |  |  |  |  |  |  |  |  |  |  | x |  |  | 1 |
| d469 | Walking and moving, other specified and unspecified |  |  |  |  |  |  |  |  |  | x |  |  |  |  |  |  |  |  |  |  |  |  |  |  |  |  |  |  |  |  |  |  |  |  | 1 |
| d475 | Driving |  |  |  |  |  |  |  |  |  | x |  |  | x |  |  |  |  |  |  |  |  |  |  |  |  |  |  |  |  |  |  |  |  |  | 2 |
| d498 | Mobility, other specified |  |  |  |  |  | x |  |  |  |  |  |  |  |  |  |  |  |  |  |  |  |  |  |  |  |  |  |  |  |  |  |  |  |  | 1 |
| *d5* | *SELF-CARE* | 4 | 0 | 0 | 2 | 1 | 0 | 0 | 0 | 0 | 0 | 0 | 0 | 0 | 3 | 1 | 0 | 1 | 0 | 2 | 2 | 0 | 0 | 0 | 0 | 0 | 0 | 0 | 0 | 1 | 1 | 0 | 2 | 0 | 1 | 21 |
| d510 | Washing oneself |  |  |  |  |  |  |  |  |  |  |  |  |  |  |  |  |  |  | x | x |  |  |  |  |  |  |  |  |  |  |  |  |  |  | 2 |
| d5101 | Washing whole body | x |  |  |  |  |  |  |  |  |  |  |  |  | x |  |  |  |  |  |  |  |  |  |  |  |  |  |  |  |  |  | x |  |  | 3 |
| d530 | Using toilet | x |  |  |  |  |  |  |  |  |  |  |  |  |  |  |  |  |  |  |  |  |  |  |  |  |  |  |  |  |  |  |  |  |  | 1 |
| d540 | Dressing | x |  |  |  |  |  |  |  |  |  |  |  |  | x |  |  |  |  | x | x |  |  |  |  |  |  |  |  |  |  |  | x |  |  | 5 |
| d550 | Crawling | x |  |  |  |  |  |  |  |  |  |  |  |  | x |  |  |  |  |  |  |  |  |  |  |  |  |  |  |  |  |  |  |  | x | 3 |
| d570 | Looking after one's health |  |  |  | x | x |  |  |  |  |  |  |  |  |  |  |  | x |  |  |  |  |  |  |  |  |  |  |  |  |  |  |  |  |  | 3 |
| d5700 | Ensuring one's physical comfort |  |  |  |  |  |  |  |  |  |  |  |  |  |  | x |  |  |  |  |  |  |  |  |  |  |  |  |  |  |  |  |  |  |  | 1 |
| d5701 | Managing diet and fitness |  |  |  |  |  |  |  |  |  |  |  |  |  |  |  |  |  |  |  |  |  |  |  |  |  |  |  |  | x | x |  |  |  |  | 2 |
| *d6* | *DOMESTIC LIFE* | 2 | 2 | 0 | 1 | 0 | 0 | 0 | 0 | 0 | 1 | 0 | 0 | 1 | 6 | 0 | 0 | 0 | 0 | 2 | 2 | 0 | 2 | 1 | 0 | 0 | 0 | 2 | 2 | 1 | 0 | 0 | 5 | 1 | 0 | 32 |
| d6200 | Shopping |  |  |  |  |  |  |  |  |  |  |  |  |  | x |  |  |  |  |  |  |  |  |  |  |  |  |  |  |  |  |  | x |  |  | 2 |
| d630 | Preparing meals |  |  |  |  |  |  |  |  |  |  |  |  |  | x |  |  |  |  |  |  |  |  |  |  |  |  |  |  |  |  |  |  |  |  | 1 |
| d640 | Doing housework |  |  |  |  |  |  |  |  |  | x |  |  | x | x |  |  |  |  | x |  |  | x |  |  |  |  | x |  |  |  |  | x | x |  | 8 |
| d6400 | Washing and drying clothes and garments |  |  |  |  |  |  |  |  |  |  |  |  |  | x |  |  |  |  |  |  |  |  |  |  |  |  |  |  |  |  |  |  |  |  | 1 |
| d6402 | Cleaning living area |  |  |  |  |  |  |  |  |  |  |  |  |  | x |  |  |  |  |  |  |  |  |  |  |  |  |  |  |  |  |  | x |  |  | 2 |
| d6403 | Using household appliances |  |  |  |  |  |  |  |  |  |  |  |  |  | x |  |  |  |  |  | x |  |  |  |  |  |  |  |  |  |  |  |  |  |  | 2 |
| d6408 | Doing housework, other specified | **x** |  |  |  |  |  |  |  |  |  |  |  |  |  |  |  |  |  |  |  |  |  | x |  |  |  | x | x |  |  |  | x |  |  | 5 |
| d650 | Caring for household objects |  | x |  |  |  |  |  |  |  |  |  |  |  |  |  |  |  |  |  |  |  |  |  |  |  |  |  |  |  |  |  |  |  |  | 1 |
| d6505 | Taking care of plants, indoors and outdoors | x |  |  |  |  |  |  |  |  |  |  |  |  |  |  |  |  |  |  | x |  | x |  |  |  |  |  |  |  |  |  | x |  |  | 4 |
| d6508 | Caring for household objects, specified |  |  |  |  |  |  |  |  |  |  |  |  |  |  |  |  |  |  |  |  |  |  |  |  |  |  |  | x |  |  |  |  |  |  | 1 |
| d660 | Assisting others |  |  |  | x |  |  |  |  |  |  |  |  |  |  |  |  |  |  |  |  |  |  |  |  |  |  |  |  |  |  |  |  |  |  | 1 |
| *D7* | *Interpersonal interactions and relationships* | 1 | 0 | 0 | 2 | 0 | 2 | 0 | 0 | 0 | 2 | 0 | 0 | 2 | 0 | 3 | 0 | 1 | 0 | 1 | 1 | 0 | 2 | 0 | 0 | 0 | 0 | 0 | 0 | 0 | 1 | 0 | 0 | 0 | 0 | 18 |
| d7500 | Informal relationships with friends |  |  |  |  |  |  |  |  |  |  |  |  | x |  | x |  |  |  |  |  |  | x |  |  |  |  |  |  |  | x |  |  |  |  | 4 |
| d760 | Family relationships |  |  |  |  |  | x |  |  |  |  |  |  | x |  |  |  |  |  |  | x |  | x |  |  |  |  |  |  |  |  |  |  |  |  | 4 |
| d7600 | Parent-child relationships |  |  |  |  |  |  |  |  |  |  |  |  |  |  | x |  |  |  |  |  |  |  |  |  |  |  |  |  |  |  |  |  |  |  | 1 |
| d770 | Intimate relationships |  |  |  | x |  |  |  |  |  | x |  |  |  |  | x |  |  |  | x |  |  |  |  |  |  |  |  |  |  |  |  |  |  |  | 4 |
| d7702 | Sexual relationships | x |  |  | x |  | x |  |  |  | x |  |  |  |  |  |  | x |  |  |  |  |  |  |  |  |  |  |  |  |  |  |  |  |  | 5 |
| *D8* | *Major life areas* | 0 | 0 | 2 | 4 | 0 | 0 | 2 | 0 | 0 | 2 | 0 | 0 | 0 | 0 | 2 | 0 | 1 | 0 | 1 | 0 | 0 | 1 | 0 | 0 | 0 | 1 | 0 | 0 | 0 | 0 | 0 | 0 | 1 | 0 | 17 |
| d820 | School education |  |  |  |  |  |  |  |  |  | x |  |  |  |  |  |  |  |  |  |  |  |  |  |  |  |  |  |  |  |  |  |  |  |  | 1 |
| d839 | Education, other specified and unspecified |  |  |  | x |  |  | x |  |  |  |  |  |  |  |  |  |  |  |  |  |  |  |  |  |  |  |  |  |  |  |  |  |  |  | 2 |
| d845 | Acquiring, keeping and terminating a job |  |  |  |  |  |  |  |  |  |  |  |  |  |  | x |  |  |  |  |  |  |  |  |  |  |  |  |  |  |  |  |  |  |  | 1 |
| d8458 | Acquiring, keeping and terminating a job, other specified |  |  |  |  |  |  |  |  |  |  |  |  |  |  | x |  |  |  |  |  |  |  |  |  |  |  |  |  |  |  |  |  |  |  | 1 |
| d850 | Remunerative employment |  |  | x | x |  |  | x |  |  | x |  |  |  |  |  |  | x |  | x |  |  | x |  |  |  | x |  |  |  |  |  |  |  |  | 8 |
| d855 | Non-remunerative employment |  |  | x | x |  |  |  |  |  |  |  |  |  |  |  |  |  |  |  |  |  |  |  |  |  |  |  |  |  |  |  |  |  |  | 2 |
| d859 | Work and employment, other specified and unspecified |  |  |  |  |  |  |  |  |  |  |  |  |  |  |  |  |  |  |  |  |  |  |  |  |  |  |  |  |  |  |  |  | x |  | 1 |
| d879 | Economic life, other specified and unspecified |  |  |  | x |  |  |  |  |  |  |  |  |  |  |  |  |  |  |  |  |  |  |  |  |  |  |  |  |  |  |  |  |  |  | 1 |
| *D9* | *Community, social and civic life* | 1 | 1 | 0 | 1 | 0 | 1 | 1 | 0 | 3 | 1 | 0 | 0 | 0 | 2 | 1 | 0 | 0 | 0 | 2 | 3 | 0 | 4 | 0 | 0 | 0 | 0 | 2 | 1 | 2 | 1 | 2 | 1 | 2 | 0 | 32 |
| d920 | Recreation and leisure |  |  |  | x |  | x |  |  | x | x |  |  |  |  |  |  |  |  | x | x |  | x |  |  |  |  | x |  | x | x |  |  | x |  | 11 |
| d9200 | Play |  |  |  |  |  |  |  |  |  |  |  |  |  | x |  |  |  |  |  | x |  |  |  |  |  |  |  |  |  |  |  |  |  |  | 2 |
| d9201 | Sports | x | x |  |  |  |  |  |  | x |  |  |  |  | x | x |  |  |  |  |  |  | x |  |  |  |  |  |  |  |  | x | x | x |  | 9 |
| d9204 | Hobbies |  |  |  |  |  |  |  |  | x |  |  |  |  |  |  |  |  |  |  |  |  | x |  |  |  |  |  |  |  |  |  |  |  |  | 2 |
| d9205 | Socializing |  |  |  |  |  |  | x |  |  |  |  |  |  |  |  |  |  |  | x | x |  | x |  |  |  |  |  | x | x |  | x |  |  |  | 7 |
| d9208 | Recreation and leisure, other specified |  |  |  |  |  |  |  |  |  |  |  |  |  |  |  |  |  |  |  |  |  |  |  |  |  |  | x |  |  |  |  |  |  |  | 1 |
| % |  | 19 | 9 | 6 | 13 | 1 | 12 | 6 | 0 | 9 | 12 | 0 | 0 | 9 | 29 | 16 | 1 | 4 | 0 | 12 | 14 | 1 | 16 | 6 | 0 | 0 | 13 | 9 | 12 | 4 | 4 | 3 | 19 | 10 | 12 |  |
| n | 69 | 13 | 6 | 4 | 9 | 1 | 8 | 4 | 0 | 6 | 8 | 0 | 0 | 6 | 20 | 11 | 1 | 3 | 0 | 8 | 10 | 1 | 11 | 4 | 0 | 0 | 9 | 6 | 8 | 3 | 3 | 2 | 13 | 7 | 8 |  |
