# Supplementary material for: A scoping review and mapping exercise comparing the content of patient-reported outcome measures (PROMs) across heart disease-specific scales
Source: J Patient Rep Outcomes. 2020 Jan 23;4:7. doi: 10.1186/s41687-019-0165-7 (PMC6977790; doi:10.1186/s41687-019-0165-7)
Supplement: Supplementary file 4 — Additional file 4. Overview of different aspects measured by each PROMs (n = 34) linked to ICF catgories from the component ‘Environmental factors’ stratified by heart diseases. [file 41687_2019_165_MOESM4_ESM.docx]

| Additional file 4. Overview of different aspects measured by each PROMs (n=34)* linked to ICF catgories from the component '*Environmental factors'* stratified by heart diseases | | | | | | | | | | | | | | | | | | | | | | | | | |  |  |  |  |  |  |  |  |  |  |  |
| --- | --- | --- | --- | --- | --- | --- | --- | --- | --- | --- | --- | --- | --- | --- | --- | --- | --- | --- | --- | --- | --- | --- | --- | --- | --- | --- | --- | --- | --- | --- | --- | --- | --- | --- | --- | --- |
|  |  |  |  |  |  |  |  |  |  |  |  |  |  |  |  |  |  |  |  |  |  |  |  |  |  |  |  |  |  |  |  |  |  |  |  |  |
| ICF categories | | Heart diseases in general | | | | | Cardiac arrythmia | | | | | | Heart failure | | | | | | | | | | | | | | Ischemic heart disease | | | | | | | | | n |
| ICF code | ICF title | DASI | HeartQoL | MILQ | QLI - cardic version | QLICD-CHD | AF-QoL-40 | ASTA | AF6 | AFEQT | PPAQ | QLAF | CHPchf | CaReQoL CHF | CHQ | CHD-TAAQOL | FEW16 | IDCV | HFSAS | KCCQ | LVD-36 | MSAS | MLHF | QLQ-SHF | SSQ-HF | TCM inquiry | ALWQ | APQOL | CLASP | MIDAS | QLI | QLMI-2 | SAQ | Summary Index for the Assessment of Quality of Life in Angina Pectoris | WHO Rose Angina questionnaire |  |
| *e1* | *Products and technology* | 0 | 0 | 1 | 1 | 0 | 1 | 1 | 0 | 1 | 0 | 1 | 0 | 0 | 0 | 1 | 0 | 0 | 0 | 0 | 1 | 0 | 2 | 0 | 0 | 0 | 0 | 0 | 0 | 1 | 0 | 0 | 1 | 0 | 0 | 12 |
| e1101 | Drugs |  |  |  |  |  | x | x |  | x |  | x |  |  |  | x |  |  |  |  | x |  | x |  |  |  |  |  |  | x |  |  | x |  |  | 9 |
| e155 | Design, construction and building products and technology of buildings for private use |  |  |  | x |  |  |  |  |  |  |  |  |  |  |  |  |  |  |  |  |  |  |  |  |  |  |  |  |  |  |  |  |  |  | 1 |
| e165 | Assets |  |  | x |  |  |  |  |  |  |  |  |  |  |  |  |  |  |  |  |  |  | x |  |  |  |  |  |  |  |  |  |  |  |  | 2 |
| *e2* | *Natural environment and human-made changes to environment* | 0 | 0 | 0 | 0 | 0 | 1 | 0 | 0 | 0 | 0 | 0 | 2 | 0 | 0 | 0 | 0 | 0 | 0 | 0 | 1 | 0 | 0 | 0 | 0 | 0 | 0 | 0 | 0 | 1 | 0 | 0 | 0 | 0 | 1 | 6 |
| e225 | Climate |  |  |  |  |  |  |  |  |  |  |  |  |  |  |  |  |  |  |  |  |  |  |  |  |  |  |  |  | x |  |  |  |  | x | 2 |
| e2250 | Temperature |  |  |  |  |  |  |  |  |  |  |  | x |  |  |  |  |  |  |  |  |  |  |  |  |  |  |  |  |  |  |  |  |  |  | 1 |
| e2254 | Wind |  |  |  |  |  |  |  |  |  |  |  | x |  |  |  |  |  |  |  |  |  |  |  |  |  |  |  |  |  |  |  |  |  |  | 1 |
| *e3* | *Support and relationships* | 0 | 0 | 1 | 4 | 0 | 1 | 0 | 0 | 0 | 0 | 0 | 0 | 1 | 0 | 0 | 0 | 0 | 0 | 0 | 1 | 0 | 0 | 0 | 0 | 0 | 0 | 0 | 0 | 2 | 1 | 1 | 1 | 0 | 0 | 3 |
| e310 | Immediate family |  |  |  | x |  |  |  |  |  |  |  |  |  |  |  |  |  |  |  |  |  |  |  |  |  |  |  |  | x | x | x |  |  |  | 4 |
| e315 | Extended family |  |  |  | x |  |  |  |  |  |  |  |  |  |  |  |  |  |  |  |  |  |  |  |  |  |  |  |  |  |  |  |  |  |  | 1 |
| e320 | Friends |  |  |  | x |  |  |  |  |  |  |  |  |  |  |  |  |  |  |  |  |  |  |  |  |  |  |  |  | x |  |  |  |  |  | 2 |
| e340 | Personal care providers and personal assistans |  |  |  |  |  |  |  |  |  |  |  |  | x |  |  |  |  |  |  |  |  |  |  |  |  |  |  |  |  |  |  |  |  |  | 1 |
| e355 | Health professionals |  |  | x |  |  |  |  |  |  |  |  |  |  |  |  |  |  |  |  |  |  |  |  |  |  |  |  |  |  |  |  | x |  |  | 2 |
| e398 | Support and relationships, other specified |  |  |  | x |  |  |  |  |  |  |  |  |  |  |  |  |  |  |  |  |  |  |  |  |  |  |  |  |  |  |  |  |  |  | 1 |
| *e4* | *Attitudes* | 0 | 0 | 0 | 0 | 0 | 0 | 0 | 0 | 0 | 0 | 0 | 0 | 0 | 0 | 0 | 0 | 0 | 0 | 0 | 0 | 0 | 0 | 0 | 0 | 0 | 0 | 0 | 0 | 0 | 0 | 1 | 0 | 0 | 0 | 1 |
| *e5* | *Services, systems and policies* | 0 | 0 | 1 | 1 | 0 | 0 | 0 | 0 | 1 | 0 | 1 | 0 | 1 | 0 | 1 | 0 | 0 | 0 | 0 | 0 | 0 | 0 | 0 | 0 | 0 | 0 | 0 | 0 | 0 | 0 | 0 | 1 | 0 | 0 | 7 |
| e580 | Education and training services, systems and policies |  |  | x | x |  |  |  |  |  |  |  |  |  |  |  |  |  |  |  |  |  |  |  |  |  |  |  |  |  |  |  | x |  |  | 3 |
| e5800 | Health services |  |  |  |  |  |  |  |  | x |  | x |  | x |  | x |  |  |  |  |  |  |  |  |  |  |  |  |  |  |  |  |  |  |  | 4 |
| % |  | 0 | 0 | 18 | 35 | 0 | 6 | 6 | 0 | 12 | 0 | 12 | 12 | 12 | 0 | 12 | 0 | 0 | 0 | 0 | 6 | 0 | 12 | 0 | 0 | 0 | 0 | 0 | 0 | 24 | 6 | 6 | 18 | 0 | 6 |  |
| n | 17 | 0 | 0 | 3 | 6 | 0 | 1 | 1 | 0 | 2 | 0 | 2 | 2 | 2 | 0 | 2 | 0 | 0 | 0 | 0 | 1 | 0 | 2 | 0 | 0 | 0 | 0 | 0 | 0 | 4 | 1 | 1 | 3 | 0 | 1 |  |
